# The Broad Effect of Iodine in Graves’ Hyperthyroidism and Its Relationship with the Gut Microbiota

**DOI:** 10.3390/nu18071082

**Published:** 2026-03-27

**Authors:** Elsbeth R. P. C. van Wees-Jansen, Barbara A. Hutten, Max Nieuwdorp

**Affiliations:** 1 Department of Internal Medicine, Amsterdam University Medical Center, University of Amsterdam, Meibergdreef 9, 1105 AZ Amsterdam, The Netherlands; 2 Department of Epidemiology and Data Science, Amsterdam University Medical Center, University of Amsterdam, Meibergdreef 9, 1105 AZ Amsterdam, The Netherlands; 3 Amsterdam Gastroenterology Endocrinology Metabolism Research Institute, 1105 AZ Amsterdam, The Netherlands; 4 Personalized Medicine and Quality of Care, Amsterdam Public Health Research Institute, 1081 BT Amsterdam, The Netherlands; 5 Diabetes & Metabolism, Amsterdam Cardiovascular Sciences Research Institute, 1105 AZ Amsterdam, The Netherlands; 6 Diabeter Center Amsterdam, 1066 EC Amsterdam, The Netherlands

**Keywords:** gut microbiome, GREAT+, diet, gut microbiota–thyroid axis, lipid profile, noncommunicable, Basedow, ophthalmopathy, metabolomics, butyrate, SCFA, gut metabolites, phylum

## Abstract

Thyroid disorders are among the most common endocrine disorders worldwide and are classified as noncommunicable diseases. These disorders are associated with significant morbidity, impaired quality of life, and considerable socioeconomic burden. Like other noncommunicable diseases, thyroid disorders arise from complex interactions between genetic susceptibility and environmental factors, including diet and lifestyle. Despite growing interest in lifestyle-based approaches to noncommunicable disease prevention and management, thyroid disorders have received comparatively limited attention in this context. Graves’ disease, the most common cause of hyperthyroidism, is a relevant condition for exploring dietary interventions. Current treatment strategies—anti-thyroid drugs, radioactive iodine and thyroidectomy—have remained largely unchanged for decades. Long-term remission following drug therapy is achieved in no more than approximately 50% of patients, while all treatment modalities carry potential adverse effects. These limitations underscore the need for alternative or adjunctive therapeutic strategies. Iodine intake plays a central role in thyroid hormone synthesis. Indeed, observational studies have shown inverse associations between iodine intake and remission rates, as well as achievement of euthyroidism, medication requirements and thyroid autoantibody titers. These findings suggest that dietary iodine restriction may enhance treatment efficacy and reduce medication-related risks. Beyond its direct effects on thyroid hormone synthesis, iodine may influence Graves’ disease through indirect mechanisms involving the lipid profile and the gut–thyroid axis. Autoimmune thyroid diseases are associated with a dyslipidemic profile and with gut microbiota dysbiosis; the latter characterized by increased potentially pathogenic bacteria and reduced beneficial bacteria such as *Lactobacillus* and *Bifidobacterium*.

## 1. Introduction

Growing international concern about the affordability of healthcare has led to increasing interest in lifestyle and nutritional interventions to influence health and disease, not only preventively but also curatively, with greater emphasis on patient autonomy [1]. Noncommunicable diseases (NCDs) are mainly investigated in this context, with a special focus on NCDs with the highest mortality: cardiovascular diseases, cancer, chronic respiratory diseases and diabetes [2]. NCDs are acquired through a combination of genetic, behavioral and environmental factors [2,3]. Unhealthy or otherwise unfitting dietary and lifestyle patterns increase the risk of developing NCDs [2,4]. Furthermore, NCDs are placing a growing social and economic burden on society [5,6,7], but many of them can be prevented by adjusting diet and/or lifestyle [8,9,10]. Thyroid disorders, which are also classified as NCDs [11], currently do not receive sufficient attention in nutrition and lifestyle options, despite being among the most prevalent endocrine disorders alongside diabetes [12] and a major cause of morbidity [13]. Global estimates of the prevalence of thyroid disorders ranges from 0.2% to 10% [12,14,15]. Prevalence varies widely and depends on the geographical area. The socioeconomic consequences of thyroid disorders are substantial [14,16,17,18] and the impact on labor productivity is significant [18]. Thyroid disorders and NCDs share a bidirectional relationship [13]; if one has a thyroid disorder, this is a risk factor for developing another NCD and vice versa. This makes it all the more necessary to investigate whether there are thyroid disorders that can be favorably influenced by a biologically plausible diet or lifestyle intervention. If NCDs in general can be positively influenced by diet or lifestyle modification, then thyroid disorders may also be modifiable.

**Figure 1:**
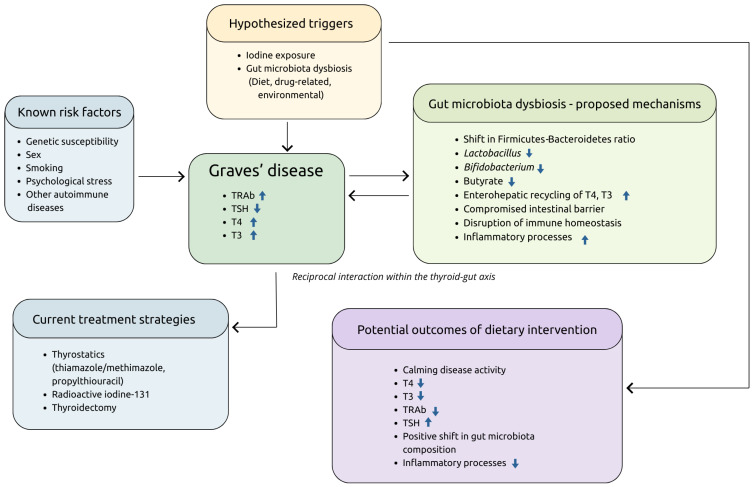
Known risk factors, hypothesized triggers and treatment options in Graves’ disease.

Figure 1 provides a concise overview of the various factors discussed in further detail in this review.

### Graves’-Basedow’s Disease

One of the thyroid disorders for which further research into lifestyle and dietary treatment options is important is Graves’–Basedow’s disease (GD), an autoimmune form of hyperthyroidism, characterized by an overactive thyroid gland caused by thyroid-stimulating antibodies (TSAb) targetting the TSH receptor (thyroid-stimulating hormone) [19]. No dietary treatment guideline is available for GD, although it is the most common cause of hyperthyroidism worldwide [20]. The lifetime risk of developing GD varies significantly and depends on a person’s environment, with estimates ranging from 0.8% to 3% [20]. Women have a 5–10-fold higher risk of developing GD. GD can occur across all age groups, with a particularly higher incidence in women aged 40 to 60 years [21,22]. Since the 1940s, the treatment of GD has not changed substantially. There is still no consensus on the optimal treatment and practice variation is wide [23,24,25,26]. The current preferred treatment for diagnosed GD consists of (medicinal) treatment with oral thyreostatics (antithyroid drugs, ATDs) for 12–18 months, after which treatment is discontinued. The probability of remission following initial treatment with ATDs is currently limited to a maximum of 50% [27,28,29,30]. Much lower remission rates are also mentioned. If relapse follows, thyreostatics are tried again for 12–18 months and if this fails again, treatment with radioactive iodine (RAI) or removal of the thyroid gland (thyroidectomy) can be considered. Both drug and non-drug treatments have potential (serious) side effects and complications. Potential side effects of ATDs include allergic reactions, muscle and joint discomfort, agranulocytosis (due to acute bone marrow depression) and acute pancreatitis. Complications of RAI treatment include salivary gland damage, taste changes, and permanent hypothyroidism, requiring lifelong thyroid hormone medication. Complications of thyroid removal include permanent hypocalcemia, permanent hypothyroidism (both of which require lifelong medication) and hoarseness due to damage to the vocal cord nerve. A large observational cohort study reported a dose–response association between radioactive iodine (RAI) treatment and mortality from solid cancers, including breast cancer [31]. Although earlier analyses and subsequent studies have not consistently confirmed a significant increased overall cancer mortality risk following RAI therapy [32,33], the findings generated discussion regarding long term safety and highlighted the importance of continued evaluation of current treatment strategies.

Furthermore, RAI treatment may cause a significant increase in TSH receptor antibodies, which may remain elevated for years [34]. In Graves’ ophthalmopathy (GO), an *eye* disease associated with Graves’ *thyroid* disease, high TSH receptor antibodies are undesirable due to their association with disease severity [35].

RAI in the treatment of GD has been associated with the worsening of pre-existing GO, as well as with de novo occurrence of GO, presumably due to the increase in TSH receptor antibodies [36,37,38,39,40]. For this reason, current guidelines recommend avoiding RAI in GD patients with active moderate-to-severe GO, and ATDs are preferred [40]. In patients with mild GO, RAI may still be considered, although glucocorticoid prophylaxis is generally recommended to reduce the risk of progression. For Graves’ *thyroid* disease, this relative contraindication is, of course, undescribed, because RAI ís one of the treatment options.

## 2. Iodine Intake in Graves’ Disease

There are indications in the scientific literature that iodine intake appears to have a negative effect in patients with hyperthyroidism, often caused by GD. However, there are no extensive trials available on this subject. As early as 1973, results of an observational study showed an inverse relationship between remission rates in GD and the population iodine intake [41]; the higher the iodine intake, the lower the probability of remission. Several other observational studies showed an association between iodine intake and the achievement of euthyroidism and remission as well [27,42,43]. Moreover, antibody titer [44] and thyreostatic dose were found to be lower in people who had less iodine in their diet [28,45,46,47,48]. A lower dose of thyroid-inhibiting medication has a lower risk of side effects than a higher dose [26]. Therefore, it is reasonable to hypothesize that limiting iodine intake may reduce thyroid hormone production in GD in the relatively short term. When using a low iodine diet in the treatment of GD, potentially a lower dose of medication would be needed for a shorter duration, thus reducing the risk of side effects and actually increasing the chances of achieving remission. Destructive treatments such as RAI treatment and thyroidectomy would thus be avoided, as well as the associated serious complications [49].

### Iodine Fortification Programs

To prevent thyroid disease due to iodine deficiency, national iodine fortification programs have been instituted [50]. Iodine is a critical component required for the synthesis of the thyroid hormone [51]. Fortification programs ensure the intake of iodine through basic foods such as bread, salt and dairy products and have played an important role in reducing iodine deficiency at a population level. Iodine deficiency can result in reduced thyroid hormone production, leading to hypothyroidism, which has been associated with various adverse health outcomes. In particular, insufficient maternal thyroid hormone availability during pregnancy directly affects fetal brain development and is associated with an increased risk of neurological and neurocognitive disorders in offspring [52]. When hypothyroidism is caused by inadequate iodine intake, it can be prevented by ensuring sufficient iodine consumption. Iodine fortification programs are therefore a textbook example of nutritional healthcare.

However, the introduction of these programs seems to have a knock-on effect on part of the population, potentially leading to the manifestation of thyroid disorders in susceptible individuals, thereby increasing the incidence of hyperthyroidism, among others. Hyperthyroidism induced by excessive iodine exposure is discussed in the Dutch guideline for thyroid dysfunction as well; euthyroid patients with this susceptibility are considered vulnerable to excessive iodine in developing overt hyperthyroidism [53]. In 2006, an article was published on the incidence increase in hyperthyroidism following the introduction of the iodine fortification program in Denmark in 1998 [54]. In younger people, the cause appeared to be mainly undetected GD. Due to the previous iodine deficient state, GD was less likely to manifest itself before iodine fortification.

As in Denmark, the introduction of iodine fortification led to an increase in the number of cases of hyperthyroidism in many countries, with an initial rise in the first few years after fortification introduction, followed by a decline [55,56].

However, the findings in these studies were inconsistent. In one study, the decrease in incidence was evenly distributed across both toxic (multi)nodular goiter and GD [55], while in another study, the decrease was only present in toxic (multi)nodular goiter and the incidence of GD remained high [56]. Yet, the first study did discuss the possibility that insufficient distinction had been made during diagnosis between toxic (multi)nodular goiter and GD, resulting in an inaccurate report on the decline in GD after iodine fortification introduction. It is important to bear this difference in mind, as this distinction is more often not made [57], even though the mechanism behind the development of hyperthyroidism as a result of iodine exposure after a period of iodine deficiency appears to be different from that in cases of underlying susceptibility to Graves’ disease. Also in the case of Denmark, six years after iodine fortification introduction the incidence of Graves’ disease remained 35% higher than before. This suggests that autoimmunity may play a role. Studies have reported a potential association between iodine and thyroid autoimmunity, both in experimental animal models and in observational studies in humans, particularly in individuals with an underlying susceptibility [58,59]. Autoimmunity resulting from iodine intake in the context of hypothyroidism has been written about regularly [60,61]. Nevertheless, with regard to hyperthyroidism, the literature is less clear and further research is needed.

## 3. Mechanism of Action Antithyroid Drugs

Zooming in on one of the current treatment alternatives, ATDs, these drugs inhibit the enzyme peroxidase in the thyroid gland and thereby the *iodination* of thyroglobulin [62,63]. This reduces the synthesis of the thyroid hormones T4 and T3 at average dietary iodine intake. Through earlier mentioned iodine fortification programs, which add iodine to most of our daily foods, we now consume in general sufficient iodine which is 150 mcg a day [64,65] in (non-pregnant) adults. Therefore, a low iodine diet could help to reduce the iodination of thyroglobulin and thus reduce the excess thyroid hormones in patients with GD. It is important to mention that nowadays, a search for new foods is going on, driven by concerns about climate change and because of the aforementioned nutrition and lifestyle interventions as possible solutions for keeping healthcare affordable. As a result, *plant-based* diets are being explored more often, with positive implications for the mentioned problems. However, *animal foods* in particular are a source of iodine; the risk of iodine deficiency again becomes more present in the population than in past decades [66]. On the other hand, the very risk of people ingesting too much iodine is also increased these days, as seaweeds are increasingly being investigated and available as a food source. Seaweed is a food that is easy to grow, but has very diverse and often excessive amounts of iodine in it [67]. In short, in light of the search for other foods in which iodine content is shifting, it is important to know what diseases this affects.

## 4. Euthyroidism, Dose–Response Relation and Reaching Remission

Laurberg et al. [25] wrote about the time to achieve euthyroidism by different doses of ATDs in relation to iodine intake and excretion; the higher the iodine intake and excretion of patients was, the higher the dose required to achieve euthyroidism. This dose–response relationship may indicate causality. In addition, achieving euthyroidism seems important in achieving remission. Abraham and Laurberg argued that there is no mysterious side effect of anti-thyroid drugs that causes the remission of Graves’ disease, which has been speculated in several studies [26]. According to them, only being euthyroid as soon as possible and for a long time ensures that the disease goes into remission [29] by breaking a vicious cycle and restoring the normal state. Thus, if a dietary reduction in iodine ensures contribution to a rapid and stable euthyroid state, it could also contribute to achieving remission.

### Definition of Remission

In this context, it is also important to discuss the definition of remission. Both Wiersinga’s and Laurberg’s definition of whether GD is cured or in remission is to strive for “A state of stable TSH, FT4 and FT3 serum concentrations in the normal range in the absence of any thyroid medication” [24,25]. Thus, adherence to the definition of remission in Graves’ disease as stated in the American Thyroid Association (ATA) guidelines, where remission is defined as serum values of TSH, FT4 and Total T3 within the normal range twelve months after discontinuation of thyreostatic use. It should immediately be noted that thyroidectomy and RAI cannot therefore be included in this definition, because in both treatment options, hyperthyroidism is not exchanged for euthyroidism (without any thyroid medication), but in most cases for hypothyroidism, in which the intake of exogenous thyroid hormones is needed. The exchange of one condition for another is not considered a cure or remission in our definition.

## 5. Graves’ Ophthalmopathy, Dyslipidemia

As mentioned earlier, RAI is generally avoided in patients with moderate-to-severe GO due to the risk of an increase in antibodies, which can lead to further deterioration of the eye disease [36,37,38,39]. The use of iodine-containing contrast agents is not recommended for similar reasons, although the differentiation based on the severity of the eye disease is less clearly described [68]. Although RAI is used as a definitive treatment for Graves’ disease, its tendency to temporarily (>5 years) [69] increase TSH receptor antibodies points to a paradoxical effect of iodine exposure, suggesting that iodine may be a risk factor not only for GO but also for the underlying autoimmune process, where the antibodies in Graves’ *thyroid* disease are the same as those in Graves’ *eye* disease. However, more research is needed to fully understand this mechanism.

Furthermore, studies assume that iodine intake affects the lipid profile [70,71] and thyroid function is known to drive a dyslipidemic profile [72], a cardiometabolic risk factor. A study by Lee and Kahaly suggested elevated cholesterol as a novel risk factor for developing GO and a less favorable response to treatment with glucocorticosteroids [35]. 

The relationship between iodine intake and the lipid profile therefore also appears to be important to include in research in order to investigate possible associations.

## 6. Gut Microbiota and Gut Metabolomics in Relation to Graves’ Disease

Evidence of an interaction between the (small) intestine and the thyroid gland, the gut–thyroid or gut microbiota–thyroid axis in general [73,74,75] and, more specifically, in GD and GO [19,35,76,77,78,79,80] is showing substantial growth. Microbiota refers to the totality of microorganisms (bacteria, viruses, protozoa, fungi) present in a specific environment (e.g., the gut), at various taxonomic levels, from phylum to species. Microbiome is a broader term and includes not only microorganisms, but also their genes and aspects of the environment (e.g., gut, skin) itself [81]. Gut microbial communities can influence thyroid hormone metabolism through several mechanisms. A healthy gut microbiome maintains intestinal barrier function, reducing systemic exposure to microbial antigens [82]. Short-chain fatty acids (SCFAs), particularly butyrate, produced by microbial fermentation of dietary fiber, support intestinal barrier function by enhancing tight junction integrity, reinforcing the epithelial mucus layer and serving as a key energy source for colonocytes [83,84]. Dysbiosis compromises this barrier with subsequent low-grade inflammation and exposure to bacterial wall components, increasing the risk of autoimmune activation [82]. In addition, gut microbiota-derived metabolites, SCFA’s like butyrate, propionate and acetate, can regulate immune cell differentiation, promoting Treg development and limiting pro-inflammatory TH17 activity [82,85]. Disruption of the gut–thyroid axis, whether due to dietary factors, medication or environmental exposures, may thus predispose individuals to thyroid disease. For instance, *Bifidobacterium* depletion has been linked to goiter development via the gut microbiota–thyroid axis, suggesting that gut microbial composition directly influences thyroid pathology [85].

### 6.1. Enterohepatic Recycling

Another important role in thyroid hormone metabolism appears to be enterohepatic recycling. Enterohepatic recycling in this context refers to the process whereby thyroxine (T4) and, to a lesser extent, triiodothyronine (T3) are metabolized in the liver, excreted into the bile and transported to the intestine, where microbial deconjugation by the gut microbiota enables the reabsorption of active hormones into the portal circulation, thereby prolonging their systemic availability and half-life. The assumption of the presence of this recycling supports the existence of a gut microbiota–thyroid axis [75,86,87]. Enterohepatic recycling has incidentally been the subject of research in the context of Graves’ disease before in a different manner. By using bile acid-binding medication (such as cholestyramine and colestipol, normally used to lower LDL-C in hypercholesterolaemia) in addition to standard drug treatment with thyreostatics, the excretion of thyroxine (T4) and triiodothyronine (T3) via the feces can be increased and the reabsorption of T4 and T3 reduced, resulting in normal FT4 and total T3 serum levels being achieved more quickly [88].

### 6.2. Iodine and Gut Microbiota

Returning to iodine, studies have shown that iodine intake alters the composition of the intestinal microbiota and induces metabolic changes in the gut microbiota–thyroid axis. This has been demonstrated in Hashimoto’s disease, autoimmune hypothyroidism [89]. In this regard, gut microbiota composition might play a significant role in triggering autoimmune diseases of the thyroid gland [90] although the exact etiology has not yet been elucidated. It is not only in Hashimoto’s disease that the gut microbiota and iodine exposure appear to play a clear role. Beyond the direct effect of iodine on thyroid function in Graves’ disease, iodine intake may also influence the gut microbiota, with potential implications for immune regulation and disease activity [74,82,89]. Evidence from observational and genetic studies suggests that variations in thyroid (dys)function [85,91] and iodine exposure can alter the composition and diversity of the gut microbiota [89,92], including shifts in the Firmicutes–Bacteroidetes ratio [93]. Firmicutes and Bacteroidetes represent the dominant bacterial phyla in the human gut (70–90% of gut bacteria belong to the phyla Firmicutes and Bacteroidetes) [94,95,96] and play key roles in SCFA production and thus in immune regulation [96,97,98]. Alterations in the relative abundance of Firmicutes and Bacteroidetes may influence microbial metabolite profiles, including butyrate production, with potential implications for immune homeostasis and inflammatory processes relevant to autoimmunity. It is therefore plausible that a low-iodine diet in the treatment of GD not only reduces thyroid overstimulation but also promotes a gut microbial profile conducive to increased butyrate production, thereby supporting the ‘calming’ of GD activity.

### 6.3. Reciprocity Thyroid with Gut Microbiome

To make it even more fascinating but also more complicated, the relationship between the thyroid gland and the gut microbiome appears to be bidirectional [99,100]. Dysbiosis of the gut microbiota is associated with impaired thyroid function, but autoimmune thyroid diseases themselves are associated with an increase in pathogenic (small) intestinal bacterial strains and a decrease in beneficial gut bacteria (e.g., *Lactobacillus* and *Bifidobacterium*) [101,102] as well. The thyroid gland and the gut microbiome influence each other reciprocally [91]. Furthermore, not only directly by shifting the above-mentioned balance of different types of beneficial and pathogenic bacteria in the gut, but also indirectly by affecting the availability of iodine for thyroid hormone synthesis due tó this shift in bacterial quantities; the gut microbiota can also play a role in iodine uptake via the sodium-iodine symporter [102]. SCFAs (mainly butyrate) play a role in the expression of this symporter. A shift in the abundance of butyrate-producing bacteria, consequently alters the uptake of iodine [100,102], thus completing the circle.

Although direct evidence linking iodine-induced changes in the gut microbiota and its metabolites to disease activity in Graves’ disease remains limited and causal relationships cannot yet be established with certainty, the integration of microbiome and metabolomics in studies offers promising opportunities for future research into nutritional interventions for this autoimmune disease.

## 7. GREAT+ Prediction Model

In 2016, a prediction model was developed to estimate, based on various clinical and laboratory characteristics, the likelihood of remission in individual patients with autoimmune hyperthyroidism; GREAT+ [103,104]. To initiate appropriate treatment for an individual patient, making a correct prediction is important. However, dietary iodine intake is not included in this model. Iodine intake could add value in making correct predictions if iodine indeed plays a significant role in achieving remission or not.

## 8. Key Publications from the Field

Only a few human prospective studies on the effect of dietary iodine in GD have been conducted. It is striking, however, that in veterinary medicine, the same treatments for hyperthyroidism are used as in humans (ATDs, RAI, thyroidectomy), but that, in addition, another treatment option is offered—namely, a low iodine diet. Veterinary studies are also cited below for this reason.

- Benker et al. [105] found, in a European (human) multicenter study, that the time needed to achieve euthyroidism in treatment in Graves’ disease depended, among other things, on iodine intake. Of the patients with low iodine status, 80% achieved thyroid serum values within the normal range within 3 weeks, whereas only 40% of patients with high iodine status achieved normal values within this time.
- In his (human) study, Azizi [47] showed that, in patients with Graves’ disease, the dosage as well as the treatment duration of thyroid-inhibiting medication depended on whether or not they lived in an iodine-sufficient area. The effectiveness of the medication was superior in patients living in an iodine-poor area compared to patients living in an iodine-rich area.
- Fritsch et al. [106] found, in a randomized and blinded (veterinary) study in which only an iodine-restricted or normal iodine-containing diet was prescribed (i.e., no medication or other treatment was used), that in the experimental group, 50% of subjects were euthyroid after 12 weeks, and in the control group, 0%. Of note, dietary iodine intake in the experimental group was 10 times lower than in the control group.
- Hui et al. [107] found in retrospective case series that, of the studied population (cats) exclusively on a low iodine diet, 42% was euthyreoid after 20 days, followed by 83% of the group after 39 days. Interestingly, the group that did not achieve immediate euthyroidism after 20 days showed higher TT4 (thyroid hormone in serum) at the start of the diet.
- Pedersen et al. [54], in their (human) study on the effects of the national iodine fortification programs in Denmark, demonstrated that the incidence of iodine-induced and autoimmune hyperthyroidism increased suggesting an undesirable side effect of increased iodine intake. In young people, the cause appeared to be mainly undetected Graves’ disease.
- In contrast, Hiraiwa et al. [108], Japan, investigated the effect of a low-iodine diet on Graves’ disease in a small randomized controlled (human) study, where no effect was found. However, as the authors point out in their discussion, the study duration was only 8 weeks, which was probably too short to induce an iodine-deficient state. This is due to the fact that a healthy body, in an average iodine-containing environment, comprises an iodine supply for about 14–19 weeks [109]. Thus, to see an initial effect, the study would have had to last at least 19 weeks, but rather (clearly) longer, because the half-life of the thyroid hormone is long and the amount of hormone produced therefore decreases, only slowly.

## 9. Clinical Equipoise

Overall, iodine intake is likely to influence thyroid function in Graves’ disease through multiple mechanisms, both directly through its effect on the production of thyroid hormones and indirectly through effects on the lipid profile, antibody titers and the composition of the gut microbiota. Despite the substantial economic burden of Graves’ disease, the optimal treatment strategy for Graves’ hyperthyroidism remains unclear [23,24,25]. Moreover, quality of life outcomes are often suboptimal [49,110], and long-term remission following initial treatment with anti-thyroid drugs is achieved in no more than approximately 50% of patients [27,28,29,30]. These limitations highlight the need to explore alternative or adjunctive therapeutic strategies, preferably based on dietary or lifestyle interventions, in line with the current paradigm shift and toward more patient-centered care. Furthermore, weight gain following treatment for hyperthyroidism is increasingly recognized as one of the factors negatively impacting long-term quality of life in patients with Graves’ disease [111,112]. Although the mechanisms are not yet fully understood, nutritional factors, including iodine intake and interactions with the gut microbiome, may be modifiable factors that warrant further investigation.

In randomized veterinary studies, dietary iodine restriction was found to be as effective as pharmacological therapy in normalizing serum thyroid hormone levels [107,113,114,115,116]. Importantly, no harmful side effects were observed; on the contrary, a protective effect on renal function was reported. In human medicine, a low-iodine diet may represent an effective and efficient complement to medicinal thyreostatic treatment as well. However, well-conducted randomized controlled trials in humans are lacking. Consequently, the clinical effectiveness of a (temporary) low-iodine diet in patients with Graves’ disease remains uncertain. The current evidence therefore supports the presence of clinical equipoise and provides a clear rationale for the design and conduct of a clinical trial. Potential benefits of a low-iodine diet include improvements in quality of life, mediated by reduced requirements for anti-thyroid drug dosing and a lower risk of treatment-related adverse effects. In addition, dietary intervention may promote self-management, reduce dependence on specialized medical care and increase patient autonomy. In this context, a Cochrane review has previously emphasized the need for further research into quality-of-life outcomes in patients with Graves’ disease [49]. An additional advantage of dietary intervention is that, once efficacy has been established, implementation in routine clinical practice would not require complex or time-consuming procedures, allowing rapid translation to patient care. The risks associated with a low-iodine diet also appear limited. Hypothyroidism represents the primary potential adverse effect but can be readily mitigated through regular monitoring of thyroid function using blood tests that are already part of standard care. As treatment complexity decreases, a shift from secondary to primary care may become feasible, with associated cost savings. Although definitive conclusions regarding the efficacy of a low-iodine diet in Graves’ disease cannot yet be drawn, and a dietary intervention may not be suitable for all patients (as adherence and proper understanding are essential), it aligns well with the principles of personalized medicine [117]. Exploring a low-iodine diet as an alternative to or in combination with thyreostatic therapy therefore appears both appropriate and justified. To this end, we have initiated the ELODI study (Effect of a low iodine diet on remission and quality of life in newly diagnosed patients with Graves–Basedow disease, registered at ICTRP; NL-OMON58211). With the ELODI study, we will focus not only on the direct relationship between iodine intake and thyroid function regulation with concomitant ATD use in Graves’ disease, but also on the gut microbiome, its gut metabolites, the lipidemic profile and on the GREAT+ prediction in these patients in relation to iodine intake.

## Data Availability

No new data were created or analyzed in this study. Data sharing is not applicable to this article.
